# Lymphocyte senescence in COPD is associated with decreased histone deacetylase 2 expression by pro-inflammatory lymphocytes

**DOI:** 10.1186/s12931-015-0287-2

**Published:** 2015-10-24

**Authors:** Greg Hodge, Hubertus Jersmann, Hai B. Tran, Eugene Roscioli, Mark Holmes, Paul N. Reynolds, Sandra Hodge

**Affiliations:** Lung Research, Hanson Institute and Department of Thoracic Medicine, Royal Adelaide Hospital, Adelaide, South Australia Australia; Department of Medicine, University of Adelaide, Adelaide, South Australia Australia

**Keywords:** Lymphocyte senescence, COPD, HDAC2, CD28nullCD8+ T and NKT-like cells, IFNγ and TNFα

## Abstract

**Background:**

Histone acetyltransferases (HAT) and histone deacetylases (HDAC) are enzymes that upregulate and down-regulate pro-inflammatory gene transcription respectively. HDAC2 is required by corticosteroids to switch off activated inflammatory genes and is reduced in lung macrophages in COPD. We have shown that COPD patients have increased steroid resistant CD28null (senescent) pro-inflammatory T and NKT-like peripheral blood cells (particularly CD8+ subsets) and we hypothesized that these changes would be associated with a loss of HDAC2 from these senescent pro-inflammatory lymphocytes.

**Methods:**

Blood was collected from 10 COPD and 10 aged-matched controls. Intracellular pro-inflammatory cytokines, IFNγ and TNFα, and expression of CD28, HDAC2 and HAT, were determined in lymphocyte subsets in the presence of ± 5 mg/ml theophylline (HDAC2 activator), 10 μM prednisolone and 2.5 ng/ml cyclosporine A (immunosuppressant), using flow cytometry.

**Results:**

There was a loss of HDAC2 from CD28null CD8+ T and NKT-like cells in COPD. There was a significant negative correlation between HDAC2 expression and the percentage of CD28null CD8+ T and NKT-like cells producing IFNγ or TNFα in all subjects (eg, COPD: R = −.763, *p* < 0.001 for T-cell IFNγ). There was a synergistic upregulation of HDAC2 and associated decrease in pro-inflammatory cytokine production in CD28nullCD8+ T and NKT-like cells in the presence of 5 mg/L theophylline + 10^−6^ M prednisolone or 2.5 ng/mL cyclosporine A (CsA).

**Conclusions:**

Lymphocyte senescence in COPD is associated with loss of HDAC2 in CD28nullCD8+ T and NKT-like cells. Alternative treatment options such as combined theophylline with low-dose CsA, that inhibit these pro-inflammatory cells, may reduce systemic inflammation in COPD.

## Background

Chronic obstructive pulmonary disease (COPD) is a leading cause of death world wide and existing treatments, such as anti-inflammatory corticosteroids, have no proven disease modifying effect [1]. The mechanisms underlying this resistance are largely unknown, particularly in lymphocytes [2]. We have reported increased production of pro-inflammatory cytokines and expression of cytotoxic mediators granzyme b and perforin in peripheral blood CD8+ T cells in the peripheral blood and lungs [3] of current and ex-smoker COPD patients compared to healthy smokers and never-smokers [4].

Our research has focused on identifying the lymphocyte subset/s resistant to current therapeutics and we have made several important discoveries. We have shown that COPD is associated with increased CD28nullCD8+ senescent cells in the peripheral blood of both current and ex-smoker COPD subjects, and showed these cells are more cytotoxic/pro-inflammatory than CD8 + CD28+ cells [5]. It has been shown that smoking enhances telomere shortening and senescence in circulating lymphocytes which have a limited proliferative capacity [6]. Recently we also showed NKT-like and NK cells were increased in bronchoalveolar lavage (BAL) of COPD patients, associated with increased cytotoxicity by both cell types [7]. CD8 + CD28null NKT-like cells have been shown to be more pro-inflammatory and cytotoxic than CD8 + CD28+ NKT-like cells in other pro-inflammatory lung diseases [8].

Our research identified increased levels of drug efflux pump, Pgp-1 in peripheral blood cytotoxic/pro-inflammatory T and NKT-like lymphocyte subsets [9] although there were no changes between CD28null and CD28+ subsets suggesting other causes were responsible for conferring steroid resistance in these lymphocyte subsets. In this regard, we recently we showed these CD28nullCD8+ T-cells have reduced levels of glucocorticoid receptor (GCR) [10]. While this may help explain their resistance to steroid treatment, there may be other factors involved.

Histone acetyltransferases (HAT) and histone deacetylases (HDAC) are enzymes that up-regulate and down-regulate pro-inflammatory gene transcription respectively [11]. HDAC2 is required by corticosteroids to switch off activated inflammatory genes and has been shown to be reduced in lung macrophages in COPD [11].

We hypothesized that levels of HDAC2 may also be decreased in peripheral blood CD28nullCD8+ T and NKT-like lymphocyte subsets in patients with COPD and conversely, levels of HAT may be increased in these subsets.

To investigate this hypothesis, we determined whether peripheral blood CD28null T cells (particularly CD8+) and NKT-like cells from COPD patients express reduced levels of HDAC2 and/or increased HAT and whether loss of HDAC2 (and/or increased HAT) is associated with increased expression of cytotoxic mediators or pro-inflammatory cytokines. Low dose theophylline has been reported to increase levels of HDAC2 in lung macrophages and reduce inflammatory gene expression [12]. We therefore also investigated the effect of theophylline and immunosuppressant, cylclosporin A (CsA) (which we had previously shown reduced levels of Pgp-1 [9]) in combination with the corticosteroid, prednisolone, on HDAC2 and associated pro-inflammatory cytokine expression by lymphocyte subsets.

## Methods

### Patient and control groups

COPD volunteers were specifically recruited for the study and informed consent obtained. There was no exacerbation of COPD for 6 weeks prior. Subjects with other co-existing lung disease or malignancy or aged greater than 75y were excluded. Ethics approval was obtained from the Royal Adelaide Hospital and the experiments were conducted with the understanding and the written consent of each participant. COPD was diagnosed using the GOLD criteria with clinical correlation (mild COPD: FEV1/FVC < 70 % but FEV1 ≥ 80 % predicted; moderate COPD FEV1 50 % ≤ 80 % predicted, severe COPD FEV1 30 % ≤ 50 % predicted, very severe COPD FEV1 < 30 %) [13]. Blood was collected from 10 patients with COPD (Table 1) all of whom were ex-smokers (at least one year) with an average of 37 pack years. No patients were receiving oral GCS.

**Table 1:** Demographic details of the COPD and control group.

Subjects	Controls	COPD
No. of subjects	10	10
Age (years)	49 (±8)	58 (±16)*
FEV1, % pred	108.4 (±9)	60.1 (±20)*
FEV1, % FVC	96 (±12)	58 (±15)*
Male/Female	8/6	6/4

Data showing mean ± SEM

*FEV1* forced expiratory volume in 1 second, *FVC* forced vital capacity,

*P < 0.05 compared to controls

Blood was also obtained from 10 aged-matched non-smoking volunteers (Table 1) with normal lung function. These were healthy, recruited volunteers with no history of airways disease. All subjects underwent spirometry as part of their routine clinical assessment. Venous blood was collected into 10 U/mL preservative free sodium heparin (DBL, Sydney, Australia), and maintained at 4 °C until processing. All patients were submitted to the same protocol and analysis performed retrospectively.

### HDAC2, HAT, granzyme b and perforin expression in T and NKT-like cell subsets

Histone acetyltransferases (HAT) and histone deacetylases (HDAC) are enzymes that up-regulate and down-regulate pro-inflammatory gene transcription respectively [11]. We have previously shown peripheral blood T and NKT-like cells express increased cytotoxic mediators perforin and granzyme b with increased cytotoxic function (4,7) but it is unknown which subsets express the greatest potential cytoxicity to lung epithelial cells [14]. To determine expression of HDAC2, HAT, and cytotoxic mediators granzyme b and peforin in CD8+ and CD8- T and NKT-like cells, aliquots of blood were added to FACS tubes and red blood cells were lysed using FACSLyse (BD Biosciences, Sydney, Australia) as described previously [4]. After 10 min, tubes were centrifuged at 300 × g for 5 min and leucocytes permeabilised using FACSPerm (BD) as previously reported [4]. Cells were then washed with wash buffer (0.5 % BSA in Isoflow (Beckman Coulter, Sydney, Australia)). After centrifuging and decanting supernatant, 5 μL of appropriately diluted anti- HDAC2 (clone Y461, Merck Millipore, Sydney, Australia) or acetylated lysine antibody (Cell Signaling Technology, Sapphire Bioscience, Sydney, Australia) was added for 15 min in the dark at RT. Cells were washed with wash buffer as above and 5 uL appropriately diluted goat anti rabbit IgG1 PE (Invitrogen, Sydney, Australia) was added for 15 min. Following washing of cells, appropriately diluted monoclonal antibodies to perforin FITC (eBiosciences, Sydney, Australia), CD3 perCP.CY5.5 (BD), CD28 PECY7 (BD), CD56 APC (Beckman Coulter, Sydney, Australia), CD8 APC.CY7 (BD), granzyme B V450 (BD) and CD45 V500 (BD) were added for 15 min in the dark at RT. After washing cells in wash buffer, centrifugation and decanting, cells were analyzed within 1 h on a FACSCanto II flow cytometer using FACSDiva software (BD). Samples were analyzed by gating lymphocytes using CD45 staining versus side scatter (SSC). A minimum of 350,000 low SSC events were acquired in list-mode format for analysis. T cells were identified as CD3 + CD56-CD45+ and NKT-like cells identified as CD3 + CD56+ CD45+ low FSC/SSC events [7].

### HDAC2, HAT and intracellular cytokine expression in T and NKT-like cell subsets

To determine expression of HDAC2 and HAT with intracellular cytokine production in CD8+ and CD8- T and NKT-like cells, aliquots of blood were stimulated as previously reported [3] with phorbol myristate acetate (25 ng/mL) (Sigma, Sydney, Australia) and ionomycin (1 μg/mL) (Sigma) in the presence of brefeldin A (1 μg/Ml) (Sigma) and the tubes incubated in a humidified 5 % CO_2_/95 % air atmosphere at 37 °C. Preliminary experiments showed the addition of brefeldin A had no effect on HDAC2 or HAT expression in these experiments. At 16 h 100 μL 20 mM EDTA/PBS was added to the culture tubes which were vortexed vigorously for 20 sec to remove adherent cells. Red blood cells were lysed and cells were permeabilized as described previously [3]. Two mL 0.5 % bovine serum albumin (Sigma/Aldrich, Sydney, Australia)/Isoflow (Beckman Coulter, Sydney, Australia) was then added and the tubes centrifuged at 300 × g for 5 min. After decanting supernatant, Fc receptors were blocked with 10 μL human immunoglobulin (Intragam: CSL, Parkville, Australia) for 10 min in the dark at RT. Cells were stained with anti-HDAC2 or acetylated lysine monoclonal antibody as described above. Following washing of cells, appropriately diluted monoclonal antibodies to IFNγ FITC, CD3 perCP.CY5.5 (BD, Sydney, Australia), CD28 PECY7 (BD), CD56 APC (Beckman Coulter, Sydney, Australia), CD8 APC.CY7 (BD), TNFα V450 (BD) and CD45 V500 (BD) were added for 15 min in the dark at room temperature. Two mL of wash buffer was then added and the tubes centrifuged at 300 × g for 5 min. After decanting, cells were analyzed within 1 h on a FACSCanto II flow cytometer using FACSDiva software (BD). Samples were analyzed by gating lymphocytes using CD45 staining versus side scatter (SSC). A minimum of 350,000 low SSC events were acquired in list-mode format for analysis. T cells were identified as CD3 + CD45+ and NKT-like cells identified as CD3 + CD56+ CD45+ low FSC/SSC events.

### Effect of drugs on HDAC2, HAT and intracellular IFNγ expression in T and NKT-like cell subsets

Our aim was to determine the effect of methylprednisolone (10^−6^M) with a selective activator of HDAC2, theophylline (5 mg/L) [11] on HDAC2 expression and production of IFNγ and TNFα by CD8+ and CD8− T and NKT-like cells. Cyclosporin A is a Pgp-1 inhibitor and we have previously shown that pro-inflammatory cytokine production was significantly reduced in T and NKT-like cells in the presence of very low dose cyclosporine A (2.5 ng/mL) [9]. The effect of HDAC inhibitor, trichostatin A (10 ng/ml) on HDAC2 expression and IFNγ and TNFα by CD8+ and CD8− T and NKT-like cells was also investigated. To determine the effect of these drugs on HDAC2 expression in pro-inflammatory T and NKT-like cells, aliquots of blood were mixed in 10 mL sterile tubes with equal volume of RPMI 10 % FCS and incubated with drugs (and combinations) and the tubes incubated in a humidified 5 % CO_2_/95 % air atmosphere at 37 °C for 24 h. Blood cultures were then stimulated as for intracellular cytokine production as described above for 16 h. Aliquots of blood cultures were then processed as for intracellular cytokines and HDAC2, HAT, IFNγ and TNFα expression as described above.

### HDAC2 expression in CD28+ and CD28null T cells by Western Blot

PBMC were isolated from blood of a cohort of control and COPD patients by standard density gradient centrifugation and cells re-suspended at 1x10^7^ mL in RPMI 1640 medium. Following stimulation as described above, 5 μL of appropriately diluted CD3 perCP.CY5.5 (BD), CD28 PE.CY7 (BD), CD56 APC (Beckman Coulter), CD8 APC.CY7 (BD) and CD45 V500 (BD) monoclonal antibodies were added for 15 min in the dark at room temperature. Cells were washed and resuspended in 1 mL RPMI and CD28+ and CD28null, CD8+ and CD8− T cells were immediately sorted on a FACSAria flow cytometer (BD).

Equal numbers of sorted CD28+ and CD28 null T cells were lysed using M-Per mammalian cell protein lysis reagent with Halt® protease inhibitor cocktail (both Thermo Scientific, Victoria, Australia). Protein samples were quantified using the DC protein assay (Bio-Rad, Victoria, Australia), and 10 μg electrophoresed using Novex® 4–12 % gradient Bis-Tris denaturing gels (Life Technologies, Victoria, Australia) and electroblotted to Trans-Blot® Turbo nitrocellulose membrane (Bio-Rad). Membranes were blocked in 5 % diploma skim milk , washed, then incubated overnight at 4 °C with anti-human HDAC2 (1:2000), followed by a 1 h incubation at RT with horse radish peroxidase-labelled anti-mouse secondary antibody (R&D Systems, MN, USA). Chemiluminescent imaging was performed using the LAS-3000 platform, and histogram analysis performed using the Multigauge software package (both FugiFilm, Tokyo, Japan). Mouse-anti Human β-actin antibody (Sigma-Aldrich, MO, USA) was used to correct for loading error for histogram analyses.

### HDAC2 expression in CD28+ and CD28null T cells by Fluorescent Microscopy

1x10^3^ sorted CD28+ and CD28 null T cells (as described above) were added to a Cytospin 4 cytocentrifuge (ThermoFisher Scientific, Scorseby, Victoria, Australia) and centrifuged for 500 g for 5 min. Slides were air dried for 10 min and cells fixed with 2.5 % formalin in PBS for 10 min. Cytospins were treated with 1 % sodium dodecyl sulphate (SDS, Sigma Aldrich, Castle Hill, NSW, Australia) in PBS for 5 min, followed by 1 h incubation with a serum-free protein blocker (Dako A/S, Glostrup, Denmark), overnight incubation at 4 °C with 1/25 diluted HDAC rabbit monoclonal antibody (Serotec, Abacus ALS, Brisbane, Australia), then 1 hour with AF594-conjugated donkey IgG F(ab’)2 fragment polyclonal antibody to rabbit IgG (Abcam, Sapphire Bioscience, Waterloo, NSW, Australia), and counterstained with DAPI (Sigma-Aldrich). Cells were washed between incubation with 0.01 M Tris-buffered saline pH 7.5, containing 0.05 % Tween-20. Immunofluorescence was detected and imaged with a Olympus IX73 fluorescence microscope (Olympus, Notting Hill, VIC, Australia). For quantitative analysis, cells from each cytospin were photographed under a 40x objective in 8 optical fields, selected in the DAPI channel for bias prevention, the mean fluorescence intensities measured then in the AF594 channel using the ImageJ software (NIH, Bethesda, MD, USA) as previously described [15].

### Statistical Analysis

Statistical analysis was performed using the Wilcoxon sign rank test. For T-cell subsets CD28null/CD8+/CD3+/CD56−/CD45+/TNFα+/IFNγ+), a sample size of n = 10 allowed a power of 98–99.5 % for analysis. Variance was estimated from our previous studies [3–5, 7]. Correlations were performed using Spearman Rho correlation tests. SPSS software was applied and differences between groups of *p* < 0.05 considered significant.

## Results

### Increased CD28null CD8+ T and NKT-like cells in COPD patients

There was a significant increase in CD28nullCD8+ T cells in patients with COPD compared with healthy controls, but no change in CD28nullCD8− T cells (CD28nullCD8+ T: 57 ± 8.4 (33 ± 8.5); CD28nullCD8− T: 7.1 ± 3.1 (5.9 ± 4.2) for COPD patients (controls) (median ± SEM) consistent with our previous findings for CD28null T cells [5]. There was a significant increase in CD28nullCD8+ NKT-like cells in patients with COPD compared with healthy controls but no change in CD28nullCD8− NKT-like cells (CD28nullCD8+ NKT-like: 39 ± 5.9 (22 ± 6.1); CD28nullCD8− T: 8.8 ± 3.6 (7.8 ± 3.3) for COPD patients (controls).

### HDAC2 and HAT expression by CD28+ and CD28null T and NKT-like cells

**Fig. 1:**
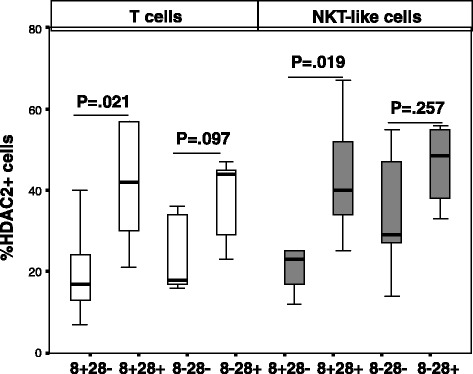
The percentage of CD28+ and CD28null (CD28-) CD8+ and CD8- T cells (clear bars) and NKT-like cells (grey bars) in patients with COPD. Data presented as box plots. There was a significant decrease in the percentage of CD28nullCD8+ T and NKT-like cells expressing HDAC2 compared with CD28+CD8- and CD28+CD8+ Tand NKT-like cells

A significantly lower percentage of CD28nullCD8+ T and NKT-like cells expressing HDAC2 in both COPD groups and controls was found, compared with CD28+ T and NKT-like cells (Data for T cell and NKT-like cell subsets from COPD group shown in Fig. 1) (data for controls not shown). There was no change in the percentage of CD28nullCD8+ or CD8− T or NKT-like cells expressing HAT in COPD patients compared with control or compared with CD28+ T and NKT-like cells (data not shown).

### Granzyme b and perforin expression by CD28+ and CD28null T and NKT-like cells

These experiments were performed to determine which subsets expressed the highest cytotoxic potential (14). There was a higher percentage of CD28nullCD8+ T cell and NKT-cells expressing perforin and granzyme b in COPD patients compared with control subjects (eg., 45 ± 13 (14 ± 12) p = .037; and 33 ± 13 (12 ± 8) p = .025 for the percentage of CD28null CD8+ T cells expressing granzyme b and perforin (median ± sd) from COPD patients (controls). There was no change in perforin or granzyme b expression in CD28 + CD8+ or CD8− T and NKT-like cells from COPD or control groups (*p* > 0.05 for all).

There was a correlation between HAT expression in CD28nullCD8+ T and NKT-like cells co-expressing perforin and granzyme b in the COPD group but not controls (eg., R = .071, P = .031 for HAT + granzyme b + CD28nullCD8+ T cells for COPD group).

### HAT, HDAC2 and IFNγ and TNFα production by CD28+ and CD28null T and NKT-like cells

**Fig. 2:**
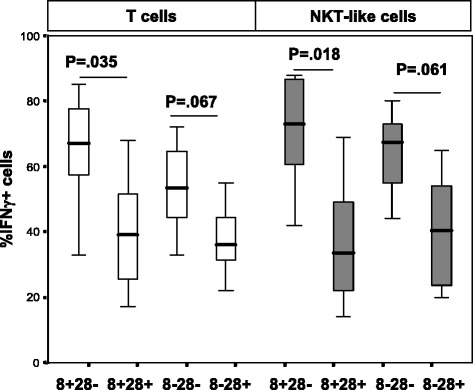
The percentage of CD28+ and CD28null (CD28-) CD8+ and CD8- T cells (clear bars) and NKT-like cells (grey bars) producing IFNγ in patients with COPD. Data presented as box plots. There was a significant decrease in the percentage of CD28nullCD8+ T and NKT-like cells expressing IFNγ compared with CD28+CD8- and CD28+CD8+ T and NKT-like cells

**Fig. 3:**
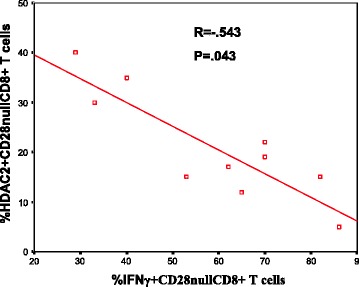
There was a significant negative correlation between the percentage of CD28nullCD8+ T cells expressing HDAC2 and producing IFNγ in COPD subjects

A significant increase in the percentage of CD28nullCD8+ T and NKT-like cells producing IFNγ and TNFα compared with CD28 + CD8+ T and NKT-like cells was noted in COPD patients and control groups (data for CD28null and CD28+ CD8+ and CD8− T and NKT-like cells producing IFNγ for the COPD group shown in Fig. 2) (data for IFNγ and TNFα production for the control group and TNFα production by the COPD group not shown). There was no correlation between HDAC2 or HAT expression and the percentage of pro-inflammatory cytokine producing T cells or NKT-like cells in COPD or control groups (data not shown). There was a negative correlation between loss of HDAC2 expression by CD28nullCD8+ T cells and the percentage of these cells producing IFNγ (Fig. 3) and TNFα (data not shown) in the COPD group but not the control group. There was a negative correlation between loss of HDAC2 expression by CD28nullCD8+ NKT-like cells and the percentage of these cells producing IFNγ (R = −.647, P = .039) and TNFα (R = −.557, P = .043) in the COPD group.

**Fig. 4:**
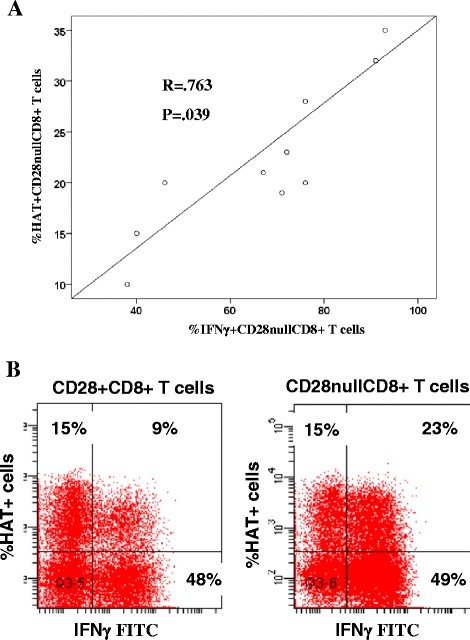
**a** There was a significant correlation between the percentage of CD28nullCD8+ T cells co-expressing HAT.IFNγ and IFNγ. **b** A representative dot plot showing an increased percentage of HAT+ CD28nullCD8+ T cells co-expressing IFNγ + (23 %) in a COPD patient compared with HAT + CD28+ CD8+ T cells (9 %)

There was a correlation between HAT expression in CD28nullCD8+ T and NKT-like cells co-expressing IFNγ (Fig. 4a). and TNFα.(not shown). A representative dot plot showing an increased percentage of HAT+ CD28nullCD8 + T cells co-expressing IFNγ + in a COPD patient compared with HAT + CD28 + CD8+ T cells is shown in Fig. 4b.

### HDAC2 expression in CD28+ and CD28null T cells by Fluorescent Microscopy

**Fig. 5:**
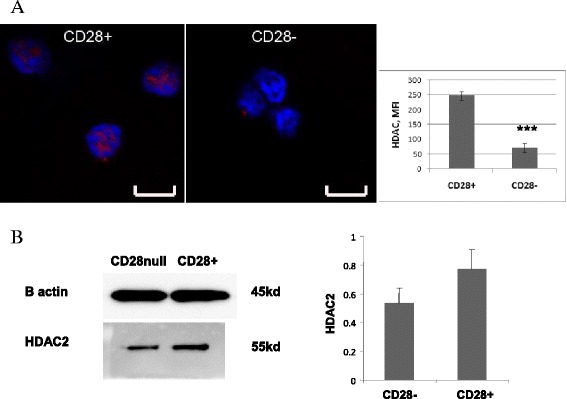
**a** Representative laser confocal images of HDAC2 staining (red) in FACS-sorted CD28null (right) and CD28+ T cells (left). Blue was DAPI counterstaining. Scale bar = 8um. The bar graph depicts results of quantitative analysis by ImageJ. Experiments were repeated 3 times, showing similar results. *** p < 0.05. **b** Representative Western Blot of equal numbers of sorted CD28+ and CD28null T cells, stained for HDAC2 expression. There was a decrease in the 55kD band corresponding to the HDAC2 in CD28null T cells compared with CD28+ T cells. Bar graph showing HDAC2 expression relative to β-actin from CD28 null (CD28-) and CD28+ T cells (mean ± sem from 3 experiments)

Sorted CD28+ and CD28null T cells were stained for HDAC2 expression. There was significant positive staining with HDAC2 in CD28+ T cells compared with CD28null T cells using fluorescence microscopy (Fig. 5b). HDAC2 staining was mainly located in the CD28+ T cell nucleus (Fig. 5a).

### HDAC2 expression of CD28+ and CD28null T cells by Western Blot

Equal numbers of FACS-sorted CD28+ and CD28null T cells were stained for HDAC2 expression by Western Blot. There was a decrease in the 55kD band corresponding to the HDAC2 in CD28null T cells compared with CD28+ T cells (Fig. 5c). HDAC2 expression relative to β-actin from CD28 null (CD28−) and CD28+ T cells (mean ± sem from 3 experiments) is shown in Fig. 5d.

### Correlation between HDAC2 by CD28nullCD8+ T cells and FEV1

**Fig. 6:**
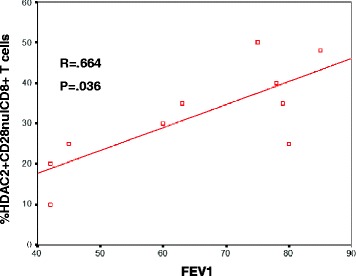
Correlation between HDAC2 expression by CD28nullCD8+ T cells with FEV1 (% predicted) in COPD subjects

There was a correlation between HDAC2 expression by CD28nullCD8+ T cells and FEV1 from the COPD group (Fig. 6) but no correlation between HDAC2 expression by any other lymphocyte subset with FEV1 (% predicted) (data not shown).

### Effect of drugs on HDAC2 and intracellular cytokine expression by CD28null CD8+ T and NKT-like cells in COPD patients

**Fig. 7:**
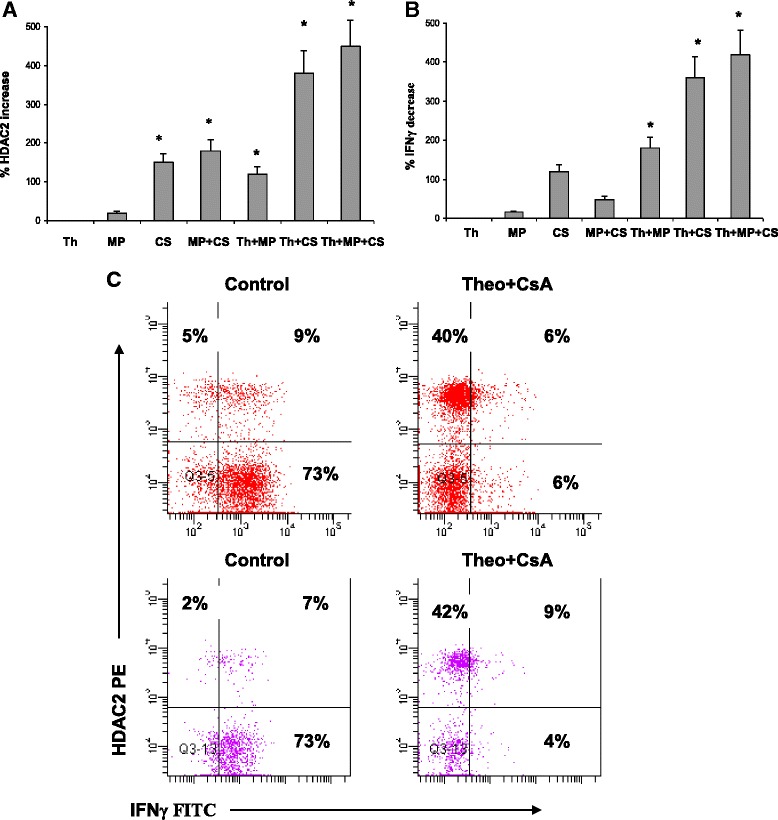
**a** Graphs showing the effect of 5 mg/ml theophylline (Th) ± 10^−6^ M prednisolone (MP) or 2.5 ng/mL cyclosporine A (CsA) or combination on the upregulation of HDAC2 (**a**) and inhibition of IFNγ production by CD28nullCD8+ T cells is shown in (**b**). There was no effect on HDAC2 or IFNγ in the presence of theophylline alone. There was significant increase in the percentage of CD28nullCD8 + T cells expressing HDAC2 in the presence of MP, CsA or combination (*p* < 0.05 for all). There was a synergistic increase in the percentage of CD28nullCD8 + T cells expressing HDAC2 in the presence of theophylline and MP or CsA or a combination. **c** Representative dot plots showing the combined effect of 5 mg/mL theophylline (Theo) and 2.5 ng/mL cyclosporine A (CsA) on the percentage of CD28null CD8+ T (top plots) and NKT-like cells (bottom plots) expressing HDAC2 and producing IFNγ. Note the significant increase in HDAC2 and significant decrease in IFNγ in both CD28null subsets in the presence of theophylline and cyclosporine A

The effect of 5 mg/ml theophylline (Th) ± 10^−6^ M methylprednisolone (MP) or 2.5 ng/ml CsA, on the upregulation of HDAC2 (Fig. 7a) and inhibition of IFNγ production by CD28nullCD8+ T cells (Fig. 7b) is shown in Fig. 7. There was no effect on HDAC2 or IFNγ in the presence of theophylline alone. There was significant increase in the percentage of CD28nullCD8+ T cells expressing HDAC2 in the presence of MP, CsA or a combination of both. There was a synergistic increase in the percentage of CD28nullCD8+ T cells expressing HDAC2 in the presence of theophylline, and MP or CsA or combination. Similar results were obtained for upregulation of HDAC2 and inhibition of IFNγ production by CD28 + CD8+ and CD8− T cells and CD28+ and CD28nullCD8+ and CD8− NKT-like cells (ie., results were similar for all T and NKT-like subsets). The presence of the HDAC inhibitor, trichostatin A (10 ng/ml), negated 80 ± 15 % (median ± SEM) of the inhibitory effect of theophylline and MP on IFNγ and TNFα by CD8+ and CD8− T and NKT-like cells. Representative dot plots showing the combined effect of 5 mg/ml theophylline and 2.5 ng/ml CsA on the percentage of CD28nullCD8+ T and NKT-like cells expressing HDAC2 and IFNγ is shown in Fig. 7c.

## Discussion

This is the first study to show that lymphocyte senescence in associated with loss of HDAC2 from CD8 + CD28null T and NKT-like cells. The loss of HDAC2 was shown to correlate with the cytotoxic/pro-inflammatory potential of these cells and importantly, disease severity in patients with COPD. HDAC2 is required by corticosteroids to switch off activated inflammatory genes [11]. We have recently shown a loss of glucocorticoid receptor (GCR) expression by these lymphocyte subsets [10]. Taken together with our current data, these findings suggest that multiple factors may be influencing steroid resistance in these cells. Interestingly, a previous study showed loss of HDAC2 by alveolar macrophages from patients with COPD without a reduction in GCR nuclear translocation [16]. In contrast, we showed similar rates of nuclear translocation of GCR in both CD28null and CD28+ T and NKT-like lymphocyte subsets, suggesting that the reduced levels of HDAC2 do not hinder GCR nuclear translocation by these cells [10]. Senescent CD28null T and NKT-like cells have been shown to be more pro-inflammatory and cytotoxic than their CD28 positive counterparts [5, 10], and exhibit a relative resistance to corticosteroids [10]. Increased pro-inflammatory CD8+ T cells in peripheral blood and lungs [3] and an increase in cytotoxic NKT-like and NK cells in the airways have been shown in COPD patients compared to healthy and never-smokers [7]. We have also identified increased CD28nullCD8+ cells in both current and ex-smoker COPD groups [5].

HDAC2 activity has been shown to be reduced in the lung parenchyma, bronchial biopsies, alveolar macrophages and peripheral blood monocytes of patients with COPD [17–19]. However, there have not been any studies identifying changes in HDAC2 in lymphocytes, particularly cytotoxic/pro-inflammatory subsets from these patients, although there has been a study that identified a reduction in total HDAC activity in PBMCs from smokers who had COPD [18]. This study showed a relationship between smoking pack-years, loss of total HDAC activity and increased serum IL-8 levels in smokers with COPD. The authors suggested this loss of total HDAC was due to a decrease in monocyte HDAC. Our findings suggest this may have been due in part to loss of HDAC2 in CD28nullCD8+ lymphocyte subsets rather than monocytes alone. In this regard our current study showed a negative correlation between the percentage of CD28nullCD8+ T and NKT-like cells producing pro-inflammatory cytokines IFNγ and TNFα and the percentage expressing HDAC2. Although the HDAC2 has been shown to be the relevant isoform for enhancement of steroid responsiveness in alveolar macrophages, measurement of HDAC1 may have proven interesting in our current study although a recent report suggests HDAC1 to be more important for maintaining CD4+ T cell lineage integrity [20] rather than CD8+ T cells.

A previous study showed no change in HAT levels in lung macrophages in patients with COPD [19] consistent with our current findings for T and NKT-like cells. However, we did find a correlation between the percentage of HAT + CD8+ T and NKT-like cells co-expressing cytotoxic mediators, perforin and granzyme b and pro-inflammatory cytokines, IFNγ and TNFα suggesting an increase in acetylation of histones in cells upregulating these pro-inflammatory cytokines with a corresponding decrease in HDAC2.

There has been a previous suggestion of an age-related or senescent-specific HDAC associated with histone H4 isoform acetylation [21], and a further study of H4 acetylation of our lymphocyte subsets may prove enlightening in this regard. HDAC activity in peripheral blood monocytes has been reported to correlate with smoker pack years in COPD patients [18]. Another important addition to our studies would be to determine whether HDAC2 levels in lymphocyte subsets are altered in smokers who have not progressed to COPD and whether there is any correlation with smoking pack years in smokers and COPD patients.

Importantly we now show these HDAC2 deficient lymphocytes are present in the systemic circulation of COPD patients. Barnes et al. proposed a spillover of cells from the lungs into the systemic circulation [2], which suggest these HDAC2 deficient cells may have originated in the lung.

Interestingly, our present study showed that a loss of HDAC2 expression by CD28nullCD8+ T and NKT-like cells was also observed in healthy control subjects, although at decreased numbers compared with patients with COPD ie., HDAC2 expression was the same in CD28null T and NKT-like cells from both subject groups. Lymphocyte senescence and GC resistance have been described in several other inflammatory conditions, such as cardiovascular disease [22], autoimmune disease [23], arthritis [24], IBD [25], aging [26] and aging with associated inflammation in COPD [27]. One could speculate that these cells may be the precursors to other inflammatory diseases. Our findings suggest that the relative GC resistance of the CD28null inflammatory lymphocytes need to be considered with any therapeutic approaches, and alternative therapies to GC may be required to avoid susceptibility to inflammatory diseases.

A previous study showed treatment with low dose theophylline upregulated total HDAC in peripheral blood monocytes and increased FEV1% predicted when combined with inhaled fluticasone propionate in patients with COPD compared with theophylline alone [28] consistent with our current findings where theophylline alone had no effect on HDAC2 but combined with prednisolone and/or very low dose cyclosporine A resulted in synergistic upregulation of HDAC2 and subsequent sensitivity to prednisolone. Furthermore, our results indicate treatment with low dose CsA with theophylline alone may increase HDAC2 and reduce inflammation and help overcome the reported adverse effects of steroids in COPD [29].

We have previously shown cytotoxic/pro-inflammatory T and NKT-like cells have increased levels of drug efflux pump, Pgp-1, and the presence of very low dose CsA, a Pgp-1 inhibitor, resulted in steroid sensitivity of these cells [9]. Taken together with our current findings this suggests that combined treatment with very low dose CsA, standard dose prednisolone and theophylline may be a drug combination of choice to target cytotoxic/pro-inflammatory lymphocytes in patients with COPD. Our ex vivo assays to study HDAC2/GCR deficient lymphocytes may identify COPD patients that would benefit from these combination of drugs. Further lymphocyte phenotyping post therapy could identify effectiveness of this therapy.

## Conclusion

In conclusion, lymphocyte senescence in COPD is associated with loss of HDAC2 in CD28nullCD8+ T and NKT-like cells. This loss is related to disease severity in COPD, thus therapies aimed at increasing HDAC2 expression in pro-inflammatory senescent lymphocytes are warranted.
